# Biochemical Characterization of the SPATE Members EspPα and EspI

**DOI:** 10.3390/toxins6092719

**Published:** 2014-09-16

**Authors:** André Weiss, David Kortemeier, Jens Brockmeyer

**Affiliations:** Institute of Food Chemistry, Corrensstraße 45, 48149 Münster, Germany

**Keywords:** EspPα, EspI, SPATE, virulence factor, EHEC, STEC, biochemical characterisation, substrate specificity

## Abstract

The activity of serine proteases is influenced by their substrate specificity as well as by the physicochemical conditions. Here, we present the characterization of key biochemical features of the two SPATE members EspPα and EspI from Shiga-toxin producing *Escherichia coli* (STEC) and enterohemorrhagic *E. coli* (EHEC). Both proteases show high activity at conditions mimicking the human blood stream. Optimal activities were observed at slightly alkaline pH and low millimolar concentrations of the divalent cations Ca^2+^ and Mg^2+^ at physiological temperatures indicating a function in the human host. Furthermore, we provide the first cleavage profile for EspI demonstrating pronounced specificity of this protease.

## 1. Introduction

The serine protease autotransporters of *Enterobacteriaceae* (SPATE) are a family of virulence factors, which are transported via the type V or classical autotransporter pathway. SPATEs harbor a serine protease motif inside their passenger domain and it is believed that SPATE proteins mediate their virulence—at least partially—via proteolytic cleavage of host proteins. The plasmid-encoded extracellular serine protease EspP belongs to the SPATE family and is present in the supernatant of Shiga-toxin producing *Escherichia coli* (STEC) and enterohemorrhagic *E. coli* (EHEC) [1,2]. Five subtypes of EspP have been described (EspPα-EspPε) [3,4] from which EspPα is clearly associated with highly pathogenic EHEC serotypes and with isolates from patients with severe disease [3,5]. EspPα has been shown to cleave porcine pepsin A, coagulation factor V, apolipoprotein A-I, the complement factors C3 and C5, and EHEC-hemolysin [1,6,7,8]. *E*. *coli* secreted protease, island-encoded (EspI) is a further member of the SPATE family and is found, like EspP, in Shiga-toxin producing *E*. *coli* [6]. Notably, this SPATE is associated with less pathogenic STEC [6,9,10,11]. It has been shown that EspI, like EspPα, cleaves porcine pepsin A and apolipoprotein A-I [6]. The physiological function of EspI remains to be elucidated.

EspI has not been characterized systematically on functional level and studies concerning functionality of EspPα have focused mainly on potential implications of this virulence factor in terms of pathogenicity of EHEC infection [1,2,3,7,8,12,13,14]. The activity of serine proteases is influenced besides the inherent substrate specificity by physicochemical aspects, such as temperature, pH, and composition of the solvent [15]. Studies addressing these biochemical aspects of EspPα and EspI are still lacking. Therefore, we present here the determination of temperature and pH-optima and the influence of divalent ions such as Mg^2+^ or Ca^2+^ on proteolytic activity, which allows to estimate to a certain extend for which environmental conditions the respective enzymes might be optimized. Furthermore, we present a cleavage profile of EspI using short chromogenic peptides as substrates to elucidate specificity of this protease.

## 2. Results and Discussion

### 2.1. Purification of EspPα and EspI

**Figure 1:**
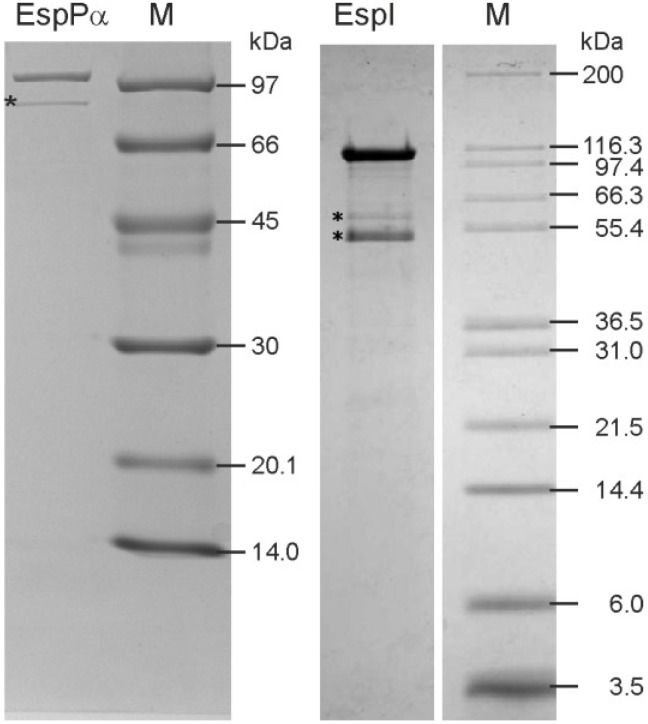
Purification of EspPα and EspI (**left**) SDS-PAGE of purified EspPα. *, EspPα autoproteolysis product; (**right**) SDS-PAGE of purified EspI. *, EspI autoproteolysis product. M = Molecular weight marker. Purity (including autoproteolysis products) of both samples was >95% as determined by densitometrical analysis of SDS-PAGE gels.

EspPα and EspI were purified from culture supernatants using ammonium sulfate precipitation and liquid chromatography. Purity was verified via SDS-PAGE (Figure 1) and the identity of autoproteolysis bands was verified by MALDI-TOF-MS (data not shown). As expected, EspPα samples showed a pronounced protein band at ~104 kDa representing the intact EspPα. Another band at ~80 kDa was identified as an autoproteolysis product of EspPα. The protein pattern of the EspI sample showed a protein band at ~110 kDa (intact EspI), as well as two autoproteolysis products of EspI at ~60 and 50 kDa, respectively. Autoproteolysis products of both proteases are still active even after long term incubation (data not shown).

### 2.2. Temperature Optimum of EspPα and EspI

**Figure 2:**
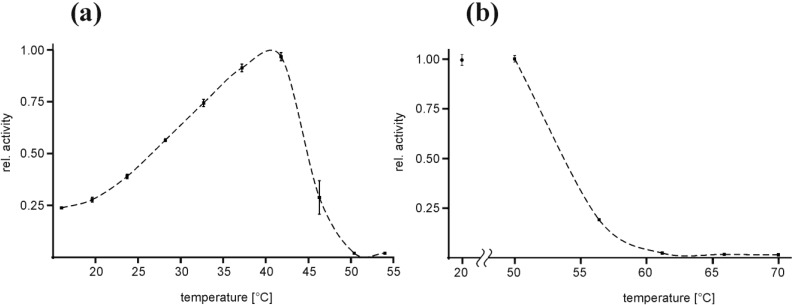
Temperature optimum and heat denaturation of EspPα (**a**) Relative activity of EspPα at varying incubation temperatures. Relative activity is normalized to *T*_opt_ at ~40 °C, *n* = 8; (**b**) Effect of 30 min pre-incubation at elevated temperatures on the activity of EspPα at 37 °C. Pre-incubation temperatures are given in the *x*-axis and the relative activity was subsequently determined at 37 °C. The negative control was incubated for 30 min at 20 °C. Relative activity is normalized to the negative control, *n* = 8.

The influence of the incubation temperature on the activity of EspPα and EspI was investigated using the chromogenic oligopeptide substrate succinic acid-alanine-alanine-proline-leucine-(para-nitroaniline) (Suc-Ala-Ala-Pro-Leu-pNA) in a temperature profile ranging from 20 °C to 55 °C for EspPα and 10 °C to 60 °C for EspI, respectively. For EspPα, maximum relative activity was observed at ~40 °C (*T*_opt_) and 50% activity values at ~26 °C and ~45 °C, respectively (Figure 2a). Activity decreased rapidly when temperature exceeded *T*_opt_ and complete loss of activity was observed at 50 °C (Figure 2a). In order to investigate if rapid loss of activity at increased temperatures is due to irreversible heat denaturation or reversible misfolding, EspPα was pre-incubated for 30 min at temperatures ranging from 50 °C to 70 °C followed by determination of residual activity at 37 °C (Figure 2b). No loss of activity was observed after pre-incubation at 50 °C, indicating that decreasing activity of EspPα in the temperature range from 40 to 50 °C is due to reversible structural alterations of EspPα. This assumption was further supported by the observation that also prolonged pre-incubation at 50 °C up to 240 min only slightly affected the residual activity of EspPα (data not shown). Increasing heat denaturation occurred at temperatures exceeding 50 °C with complete loss of activity after 30 min of pre-incubation at 60 °C or higher temperatures (Figure 2b).

**Figure 3:**
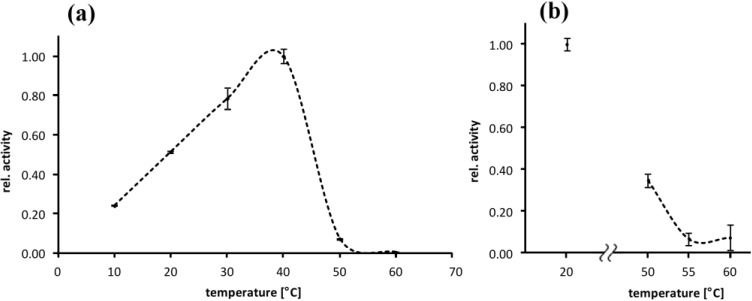
Temperature optimum and heat denaturation of EspI (**a**) Relative activity of EspI at varying incubation temperatures. Relative activity is normalized to *T*_opt_ at ~38 °C, *n* = 3; (**b**) Effect of 120 min pre-incubation at elevated temperatures on the activity of EspI at 37 °C. Pre-incubation temperatures are given in the *x*-axis and the relative activity was subsequently determined at 37 °C. The negative control was incubated for 120 min at 20 °C. Relative activity is normalized to the negative control, *n* = 3.

The temperature dependence of EspI-activity is similar to EspPα. Maximum relative activity of EspI was observed at ~38 °C (*T*_opt_) and 50% values at ~20 °C and ~45 °C, respectively (Figure 2a). EspI activity increased linearly from 10 °C to *T*_opt_. and further temperature increase resulted in rapid reduction of EspI activity with complete loss of activity at ~50 °C (Figure 3a). In contrast to EspPα, pre-incubation at 50 °C lead to significant loss of activity (residual activity < 40%) indicating EspI is more prone to heat denaturation than EspPα. Pre-incubation at 55 °C and 60 °C leads to almost complete loss of activity (Figure 3b).

### 2.3. pH Optimum of EspPα and EspI

**Figure 4:**
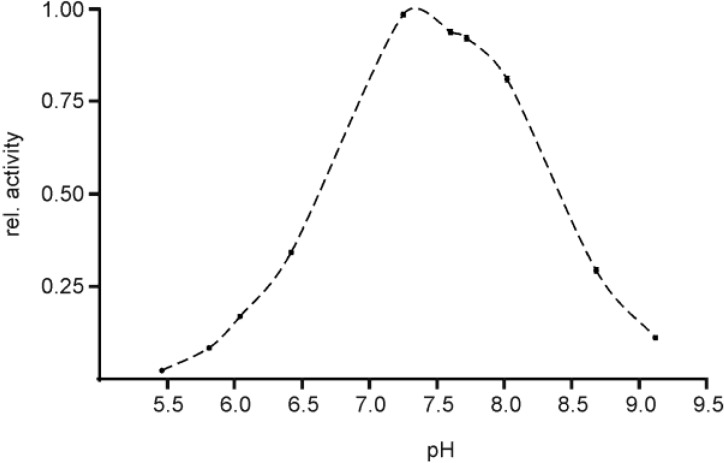
Determination of the pH optimum of EspPα. Activity of EspPα was determined in the pH range from 5.5 to 9.1. The proteolytic activity is expressed relative to pH_opt_ at ~7.4, *n* = 5.

Determination of pH dependence of EspPα activity revealed a pronounced optimum at pH values of ~7.5 and relative activity of 50% at pH 6.6 and 8.4 (Figure 4). Buffer conditions with pH values below 6.0 or above 9.0 resulted in nearly complete loss of proteolytic activity of EspPα, indicating that this protease is highly optimized for environmental conditions in a slightly alkaline milieu between pH 7 and 8.

**Figure 5:**
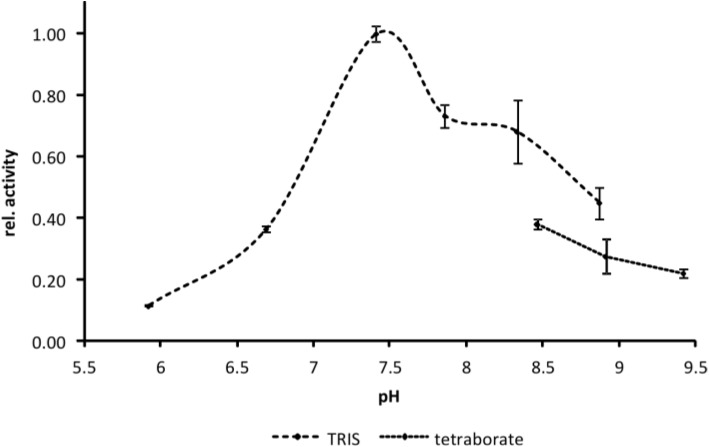
Determination of the pH optimum of EspI. Activity of EspI was determined in the pH range from 5.9 to 9.4. The proteolytic activity is expressed relative to pH_opt_ at ~7.5. Note that EspI activity in TRIS buffer is higher than in tetraborate buffer. *n* = 4.

EspI activity shows a pronounced maximum at pH ~7.5 and relative activity of 50% at pH 6.8 and 8.7. Decreasing pH values lead to rapid loss of activity while more alkaline conditions led to only slow reduction of activity (Figure 5).

### 2.4. Effect of Buffer Composition

**Figure 6:**
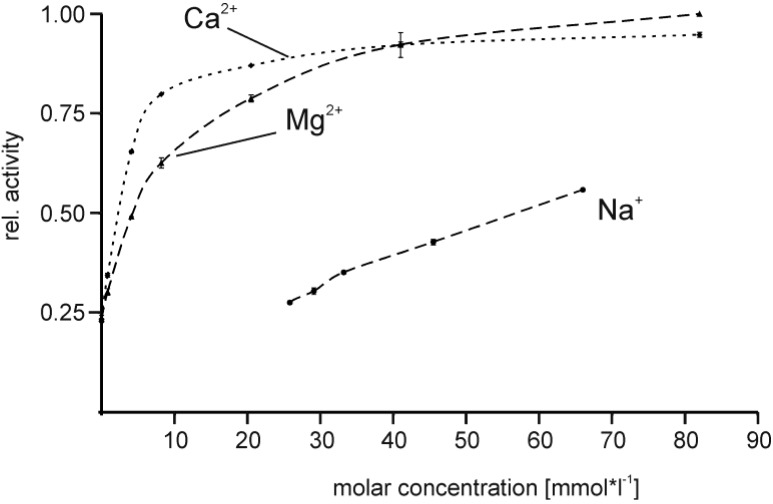
Influence of buffer composition on the activity of EspPα. Relative activity is normalized to the maximal activity observed for 82.5 mM Mg^2+^. *n* = 8.

The addition of divalent cations significantly affected proteolytic activity of EspPα. Supplementation with up to 8 mM CaCl_2_ resulted in nearly fourfold increased activity of EspPα in a dose-dependent manner when compared to the respective buffer lacking divalent cations. Further increase of the Ca^2+^ concentration resulted in plateau formation of EspPα activity, suggesting saturation with Ca^2+^ (Figure 6). The addition of MgCl_2_ led to similar effects though the activity plateau was reached at higher concentrations (Figure 6). Supplementation with NaCl resulted in moderate increase of activity in a linear correlation (Figure 6) and plateau formation at concentrations between 150 and 200 mM (data not shown).

**Figure 7:**
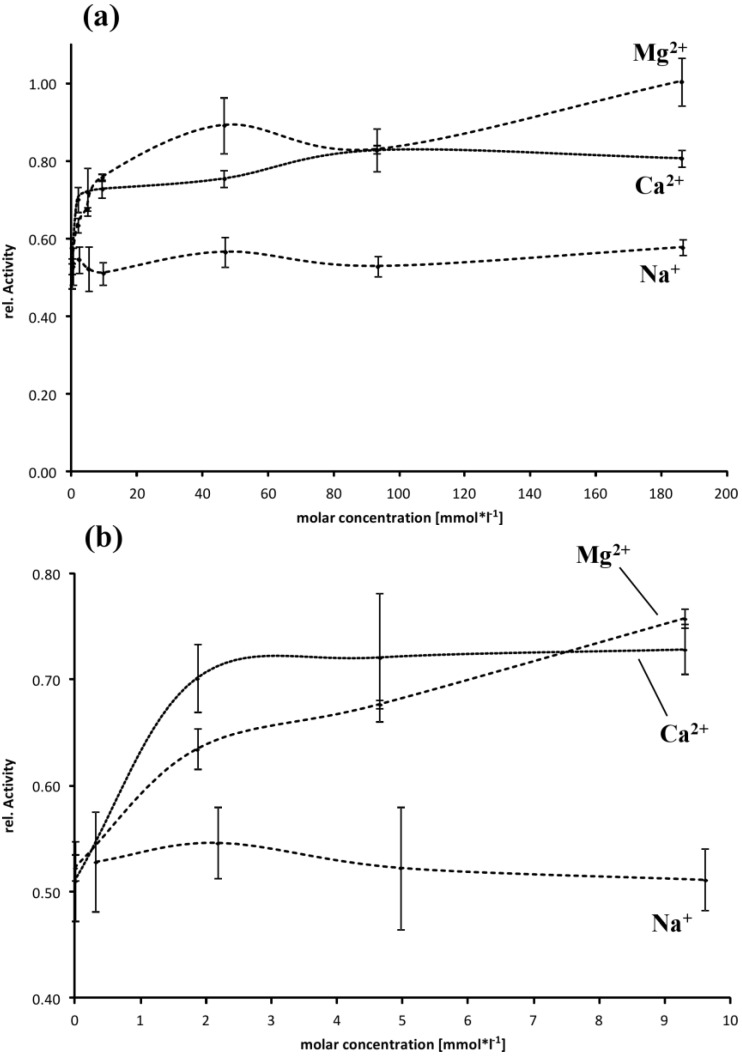
Influence of buffer composition on the activity of EspI. (**a**) Relative activity is normalized to the maximal activity observed for 186 mM Mg^2+^, *n* = 4; (**b**) Detailed view of cation concentrations in the range from 0 to 9.6 (Na^+^) or 9.3 mM (Ca^2+^, Mg^2+^).

Influence of cations on EspI activity is less pronounced compared to EspPα. Activity of EspI is independent of Na^+^ (Figure 7a) while the addition of Ca^2+^ and Mg^2+^ resulted in a slight increase in activity. More specific, EspI activity is increased ~1.4-fold by addition of ~10 mM of the divalent cations (Figure 7b). Addition of higher concentrations of Ca^2+^ or Mg^2+^ did not further increase activity (Figure 7a).

### 2.5. Cleavage Profile of EspI

**Figure 8:**
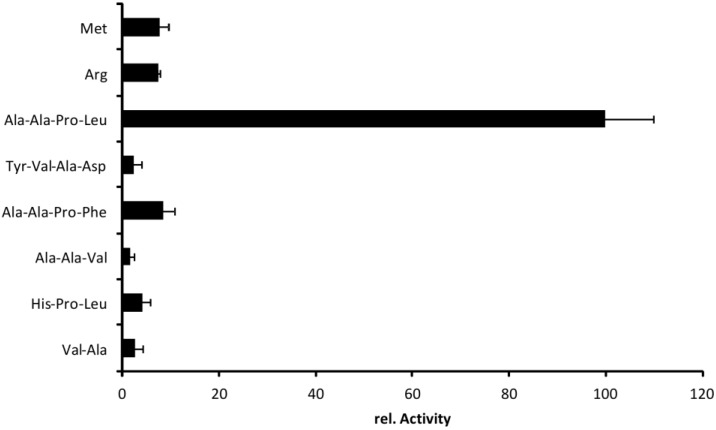
Cleavage profile of EspI. Chromogenic substrates were incubated with EspI. Activity is normalized to the maximal activity observed after incubation of Suc-Ala-Ala-Pro-Leu-pNA, *n* = 3.

Dutta* et al.* (2002) performed a substrate screening with chromogenic peptides for different SPATE members including EspPα [12]. Highest activity of EspPα was observed in this study for Succinic acid-alanine-proline-leucine-(para-nitroaniline) (Suc-Ala-Pro-Leu-pNA) and was confirmed by our group [2,3,12]. However, we did not observe cleavage of H-Arg-Arg-pNA or Bz-Arg-pNA as described in the initial study (data not shown). To elucidate substrate specificity of EspI, we incubated different chromogenic peptide substrates with EspI and changed substrate recognition sites according to the nomenclature of Schechter and Berger [16] (Figure 8). Like EspPα, EspI efficiently cleaved Suc-Ala-Ala-Pro-Leu-pNA. Modification of substrate sites P1–P4 resulted in significant reduction of relative activity. Hydrophobic amino acids other than leucine at position P1 prevented cleavage nearly completely. Also, exchange of alanine-alanine (P3–P4) to histidine (P3) significantly reduced cleavage of peptide substrates, indicating that EspI exhibits high substrate specificity and an elastase-like substrate profile. High substrate specificity has also been reported for other SPATE members [12,17], and is confirmed for EspI, which belongs to the elastase-like branch of SPATE proteases.

## 3. Experimental Section

### 3.1. Purification of EspPα and EspI

EspPα was purified from clone HB101 (WB4-5k) containing *espP* from *E*. *coli* O157:H7 strain EDL933 [1]. The inactive EspPα mutant S263A served as a negative control [2] and EspI was purified in the same way from clone DH5α/pZH4 containing *espI* from *E*. *coli* O91:H^−^ strain 4797/97 [3,6]. Strains were grown overnight in 1 L of LB broth at 37 °C with vigorous shaking. The cultures were centrifuged (6000× *g*, 30 min, 4 °C), supernatants were passed through 0.2 µm Supor machV bottle-top filters (Nalgene, Rochester, NY, USA), and the supernatant was concentrated 20-fold using Vivaflow 200 PES membrane with 50 kDa molecular weight cut off (Vivascience, Hannover, Germany) and Masterflex easy-load peristaltic pump (Cole Parmer, Vernon Hills, Chicago, IL, USA). Proteins were precipitated (3 h, 4 °C) by adding ammonium sulfate (Merck, Darmstadt, Germany) to 55% saturation. The precipitate was collected by centrifugation (6000× *g*, 30 min, 4 °C) and the pellet was dissolved. For purification of EspPα, the precipitate was dissolved in 10 mM HEPES (*N*-2-hydroxyethylpiperazine-*N*'-2-ethanesulfonic acid) buffer containing 150 mM NaCl (pH 7.4). EspPα was purified via liquid chromatography (äkta prime FPLC, GE Healthcare, Uppsala, Sweden) using HiTrap Benzamidine FF columns (GE Healthcare, München, Germany) according to the manufacturer’s instructions. For purification of EspI, the precipitate was dissolved in 20 mM Tris (tris(hydroxymethyl)aminomethane) buffer containing 50 mM NaCl (pH 6.5). EspI was purified via liquid chromatography using HiPrep 16/10 DEAE FF columns (GE Healthcare, München, Germany) and HiTrap Benzamidine FF columns according to the manufacturer’s instructions. Fractions containing EspPα or EspI were collected and concentrated with 10 kDa VivaSpin spin-down filter (Sartorius Stedim Biotech, Göttingen, Germany).

To verify purification of EspPα and EspI, proteases were separated via SDS-PAGE [18,19] and stained with Coomassie blue. Purity was determined (semiquantitatively) by densitometric analysis of Coomassie-stained SDS-PAGE gels using the Chemidoc XRS imager and the Quantity One Software package (both Biorad, Munich, Germany) Proteolytic activity was assessed via incubation of the proteases with chromogenic oligopeptide substrates. 2 µg of EspPα were incubated in a total volume of 100 µL HEPES buffer containing 150 mM NaCl and 2 mM succinic acid-alanine-alanine-proline-leucine-(para-nitroaniline) (Suc-Ala-Ala-Pro-Leu-pNA) (Bachem, Weil am Rhein, Germany) (pH 7.4) for 15 h at 37 °C. EspI cleaves the chromogenic oligopeptide substrate considerably faster. Therefore, smaller amounts of EspI were used. Since EspPα was incubated in the buffer used for its purification, the same strategy was applied for EspI. 0.2 µg of EspI were incubated in a total volume of 100 µL PBS (phosphate buffered saline, 7.0 mM Na_2_HPO_4_, 3.0 mM KH_2_PO_4_) buffer containing 100 mM NaCl and 4.5 mM KCl) and 2 mM Ala-Ala-Pro-Leu-pNA (pH 7.4) for 15 h at 37 °C. The inactive EspPα mutant S263A served as a negative control and was incubated in the same way as EspPα. Activity was analyzed by the photometrical determination of para-nitroaniline release from the chromogenic substrate in a 96-well format using the Dynex Opsis plate reader (Dynex Opsys MR, Berlin, Germany).

### 3.2. Temperature Optimum of EspPα and EspI

Samples of 2 µg of EspPα were incubated in a total volume of 100 µL HEPES buffer containing 150 mM NaCl and 2 mM of the chromogenic oligopeptide substrate Suc-Ala-Ala-Pro-Leu-pNA (pH 7.4) for 12.5 h at temperatures ranging from 16.0 to 54.0 °C. Samples of 0.2 µg of EspI were incubated in a total volume of 100 µL PBS buffer containing 100 mM NaCl and 4.5 mM KCl) and 2 mM Ala-Ala-Pro-Leu-pNA (pH 7.4) for 15 h at temperatures ranging from 10.0 to 60.0 °C. To ensure optimal temperature control, the incubation was performed in parallel in a gradient thermocycler (Biometra Gradient Cycler, Göttingen, Germany) at the respective temperatures. The negative control was stored at −20 °C during incubation. Relative activity of EspPα or EspI was analyzed subsequently as the amount of cleaved chromogenic substrate by photometrical determination of released para-nitroaniline at 405 nm in a plate reader using 96-well plates.

### 3.3. pH Optimum of EspPα and EspI

For the determination of pH optimum, 4 µg of EspPα were incubated for 15 h at 37 °C in either 166 mM potassium-phthalate buffer (pH 5.4 to 6.4) or in 166 mM Tris buffer (pH 6.0 to 9.1) with 2 mM of the chromogenic substrate Ala-Ala-Pro-Leu-pNA in a total volume of 200 µL. 0.2 µg of EspI were incubated for 15 h at 37 °C in a total volume of 100 µL with 2 mM Suc-Ala-Ala-Pro-Leu-pNA in either 150 mM Tris buffer (pH 5.9 to 8.9) or in 25 mM tetraborate buffer (pH 8.5 to 9.4) because this protease showed activity in a more alkaline environment than EspPα. The pH of the individual buffer stock solutions was adjusted with 2 M HCl or 1 M NaOH, respectively, and re-examined after addition of EspPα or EspI and the chromogenic substrate. The relative activity was analyzed by the photometrical determination of para-nitroaniline release from the chromogenic substrate in a 96-well format using the Dynex Opsis plate reader.

### 3.4. Effect of Buffer Composition

For incubation of EspPα, stock solutions each containing 100 mM CaCl_2_, MgCl_2_ or NaCl in 25 mM HEPES, pH 7.4 were diluted with 25 mM HEPES to obtain concentrations of the respective cations ranging from 0 to 82.5 mM. As the HEPES was used as Na-salt, the lowest concentration of Na^+^ was 25 mM in all experiments. In the case of EspI, stock solutions containing 200 mM CaCl_2_, MgCl_2_ or NaCl in 100 mM Tris, pH 7.4 were diluted with 100 mM Tris leading to concentrations of the respective cations ranging from 0 to 186 mM. As the purified EspI was present in PBS, the lowest concentration of Na^+^ was 0.3 mM in each experiment. These conditions allowed us to investigate a possible effect of even small amounts of Na^+^ on EspI. 2 µg of EspPα or 0.2 µg of EspI were incubated for 15 h at 37 °C with 2 mM of the chromogenic substrate Suc-Ala-Ala-Pro-Leu-pNA in the respective buffer solutions and activity was assessed by photometrical determination of released para-nitroaniline at 405 nm in a Dynex Opsys plate reader.

### 3.5. Cleavage Profile of EspI

To determine a cleavage profile for EspI, the chromogenic peptides valine-alanine-(para-nitroaniline) (H-Val-Ala-pNA), succinic acid-histidine-proline-leucine-(para-nitroaniline) (Suc-His-Pro-Leu-pNA), succinic acid-alanine-alanine-valine-(para-nitroaniline) (Suc-Ala-Ala-Val-pNA), succinic acid-alanine-alanine-proline-phenylalanine-(para-nitroaniline) (Suc-Ala-Ala-Pro-Phe-pNA), acetyl-tyrosine-valine-alanine-aspartic acid-(para-nitroaniline) (Ac-Tyr-Val-Ala-Asp-pNA), succinic acid-alanine-alanine-proline-leucine-(para-nitroaniline) (Suc-Ala-Ala-Pro-Leu-pNA), benzoyl-arginine-(para-nitroaniline) (Bz-Arg-pNA), and methionine-(para-nitroaniline) (H-Met-pNA) (all Bachem, Weil am Rhein, Germany) were dissolved in dimethylsulfoxide (DMSO). 0.2 µg EspI were incubated for 15 h at 37 °C with 2 mM of chromogenic peptide in 100 µL PBS (pH 7.4) containing 5% DMSO. Activity was assessed by photometrical determination of released para-nitroaniline at 405 nm in a BMG LABTECH FLUOstar Optima plate reader.

## 4. Conclusions

The analysis of various optima of EspPα regarding temperature, pH, and composition of the solvent suggests that this protease is seemingly well optimized for the conditions at the site of action. EspPα is expressed by EHEC during infection as indicated by the presence of anti-EspPα antibodies in the sera of patients suffering from EHEC [1]. Presumed that EspPα is expressed to act in the human host, the observed temperature optimum is with ~40 °C very close to the situation in the native environment. This observation is however no matter of course for proteases. As with enzymes in general, the activity of proteases at elevated temperatures is limited mainly by the structural stability of the protein. Rising temperatures lead to increased substrate turnover rates and thus increased enzymatic activity. As a rule, an increase in temperature of 10 °C leads to an approximately twofold elevated enzymatic activity [15]. This principle holds true as long as the native fold of the protease is sustained at the given temperature. As a consequence, proteases with more rigid structures are known, which display temperature optima far beyond their native environmental conditions [20,21,22]. Based on the data presented in this study, EspPα seems to be “as temperature-stable as necessary” but “as flexible as possible”. This assumption is supported by recent hypotheses concerning the autotransport mechanism of SPATE proteins [23]. During secretion EspPα retains a loosely folded state which is maintained by interaction with several chaperones [24,25,26,27]. After translocation through the bacterial outer membrane folding of the proteolytic domain of EspPα is initiated [24]. In addition, Jong* et al.* (2007) have shown that the related SPATE protein Hbp needs a certain degree of flexibility for the efficient translocation through the bacterial cell envelope [28]. Moreover, results of the structure-function analysis of EspPα are in accordance with these findings [2]. It is therefore likely that EspPα is optimized for proteolytic activity at physiological temperatures in the human host but has to retain in addition maximal structural flexibility to fulfill autotransport.

Optimal proteolytic activity of EspPα was observed at slightly alkaline conditions and Ca^2+^ or Mg^2+^ concentrations of approximately 8 mmol/L, suggesting that these conditions mimic best the natural environment of EspPα. Notably, the pH within the human bloodstream is constant between 7.35–7.45 and the concentrations of Ca^2+^ and Mg^2+^ are ~2.5 and ~1 mmol/L and approximately 140 mmol/L Na^+^ [29]. Investigation of the activity of EspPα suggests that Ca^2+^-binding sites within the molecule might contribute to the stability of EspPα. It has been shown for the protease family of subtilases that binding of calcium ions is essential for correct folding and stability [30]. The SPATE protein Hbp/Tsh also exhibits a Ca^2+^-binding domain [31]. Ca^2+^ does not affect the proteolytic activity which lead to the suggestion that it is important for the stability of this protease [31]. Notably, the investigation of potential calcium-binding sites within the crystal structure of EspPα (pdb: 3SZE) [32] using the WebFEATURE program [33] indicated that Ca^2+^ binding might occur at the interfacing α-helical region connecting the proteolytic domain1 and the β-helical backbone as well as in loop 165 (data not shown). Mutagenesis experiments within these regions of EspPα have shown that alterations lead to the loss of proteolytic activity and diminished autotransport, respectively [1,2]. The diminished proteolytic activity of the EspP subtypes β and δ underlies, at least in parts, the modification of the α-helical interface [3]. It is therefore tempting to speculate, that the specific alterations in the proteolytic inactive subtypes β and δ might also affect calcium-binding and thus leads to reduced structural stabilization and consequently to diminished proteolytic activity.

EspI shows, in general, similar biochemical characteristics compared to EspPα. With a temperature optimum of ~38 °C, EspI might also be optimized to act in the human host during STEC infection. Like EspPα, it rapidly loses activity at higher temperatures, demonstrating that this SPATE protease also shows certain flexibility in folding and limited temperature-stability, which might be a necessary feature for autotransport. In direct comparison to EspPα, EspI possibly needs a slightly higher degree of flexibility as demonstrated by the fact that irreversible heat denaturation occurs at lower temperatures. Presuming that the underlying secretion mechanism of EspI is identical as for EspPα [23], EspI requires a flexible structure during transport across the bacterial outer membrane. Outside of the bacterial outer membrane the flexible passenger domain of EspI might begin to fold, like EspPα, providing a part of the energy necessary for secretion. The EspI precursor, as well as the passenger domain, are slightly larger than the equivalent regions in EspPα and, therefore, might need more temperature sensitive flexible regions to facilitate autotransport. With a pH optimum at slightly alkaline conditions and increased activity at low millimolar concentrations of Ca^2+^ and Mg^2+^, EspI might, like EspPα, be suited to act in the human blood stream. It has however to be noted that the molecular basis of differences in biochemical characteristics between EspPα and EspI still remain elusive as no structure is available for EspI.

Concerning substrate specificity, it is in general believed that differences in specificity amongst SPATE proteins translate into different biological functions including pathogenicity. EspI cleaved Suc-Ala-Ala-Pro-Leu-pNA with high specificity. Exchange of amino acids at position P1 as well as at position P3/P4 strongly reduced cleavage. This high degree of specificity might be associated with specific physiological functions of this protease, which need to be elucidated in future studies.
